# Bone Marrow Mesenchymal Stem Cells Enhance the Differentiation of Human Switched Memory B Lymphocytes into Plasma Cells in Serum-Free Medium

**DOI:** 10.1155/2016/7801781

**Published:** 2016-10-31

**Authors:** Guillaume Bonnaure, Catherine Gervais-St-Amour, Sonia Néron

**Affiliations:** ^1^Hema-Quebec's Department of Research and Development, 1070 Avenue des Sciences-de-la-Vie, Québec, QC, Canada G1V 5C3; ^2^Department of Biochemistry, Microbiology and Bioinformatics, Laval University, 1045 Avenue de la Médecine, Québec, QC, Canada G1V 0A6

## Abstract

The differentiation of human B lymphocytes into plasma cells is one of the most stirring questions with regard to adaptive immunity. However, the terminal differentiation and survival of plasma cells are still topics with much to be discovered, especially when targeting switched memory B lymphocytes. Plasma cells can migrate to the bone marrow in response to a CXCL12 gradient and survive for several years while secreting antibodies. In this study, we aimed to get closer to niches favoring plasma cell survival. We tested low oxygen concentrations and coculture with mesenchymal stem cells (MSC) from human bone marrow. Besides, all cultures were performed using an animal protein-free medium. Overall, our model enables the generation of high proportions of CD38^+^CD138^+^CD31^+^ plasma cells (≥50%) when CD40-activated switched memory B lymphocytes were cultured in direct contact with mesenchymal stem cells. In these cultures, the secretion of CXCL12 and TGF-*β*, usually found in the bone marrow, was linked to the presence of MSC. The level of oxygen appeared less impactful than the contact with MSC. This study shows for the first time that expanded switched memory B lymphocytes can be differentiated into plasma cells using exclusively a serum-free medium.

## 1. Introduction

Following their activation, memory B lymphocytes differentiate into plasmablasts, namely, a preplasma like-cell, which can simultaneously proliferate and secrete antibodies [1]. Plasmablasts can quickly differentiate into short-lived plasma cells that secrete high amounts of antibodies, more than 10.000 molecules per cell per second [2, 3]. An increase in reactive oxygen species secondary to this important protein synthesis causes an oxidative stress responsible for their short lifespan [4, 5]. IgG short-lived plasma cells are known to migrate towards infection sites, while IgA populations favor mucosal tissues [6, 7]. A germinal center reaction will generate memory B lymphocytes as well as a second wave of “memory” plasma cells, which will strengthen the initial wave of short-lived plasma cells [8] that may migrate and persist in survival niches in the bone marrow [9]. To reach the bone marrow microenvironment, plasma cells need to express the protein C-X-C chemokine receptor 4 (CXCR4) [10, 11], a Transmembrane Activator, and CAML Interactor (TACI) [12, 13]. These receptors bind C-X-C chemokine ligand 12 (CXCL12) and A Proliferative Induced Ligand (APRIL) [11, 14]. IL-6 is also present and acts as prosurvival factor [11]. Furthermore, as plasma cell niches found in the bone marrow are limited in number, a competition occurs within resident cells and newly formed cells to replenish the long-lived plasma cell pool [15, 16].

Bone marrow niches involved in plasma cell survival are highly complex environment characterized by a very low level of oxygen (pO_2_), ranging from 1 to 6%, depending on blood vessel vicinity [17, 18]. These niches include several cell populations [19], such as megakaryocytes [20], basophils [21], monocytes [12], macrophages [22], dendritic cells [23], and T-cells [24]. Mesenchymal stem cells (MSC) as well as eosinophils are also present in bone marrow niches and appear to be essential for plasma cell survival [9, 25–27]. Eosinophils secrete IL-6 as well as APRIL, both promoting the maintenance and survival of plasma cells in the bone marrow for several years [28]. In the bone marrow, eosinophils are found to be highly proliferative and located in the vicinity of plasma cells [26, 27]. MSC are involved in the secretion of extracellular components and cytokines [29]. MSC secrete CXCL12 [30, 31], which is involved in plasma cell migration as well as survival in bone marrow niches [32, 33]. MSC secrete transforming growth factor beta-1 (TGF-*β*1) and TGF-*β*2, involved in B-cell homeostasis and death regulation in mouse models [34, 35]. In addition, MSC can synthetize extracellular matrix molecules such as laminin, heparan sulfate, and elastin [22, 29, 36]. As reported in a mouse model study, the ability of plasma cells to adhere to their bone marrow niche is necessary for their survival and can be impaired by anti-VLA-4 and anti-LFA treatments [37]. Besides, VCAM-1 and ICAM-1, which are natural ligands for VLA-4 and LFA, respectively, are present on MSC and are involved in cell-cell and extracellular matrix interactions [38].

The requirement for MSC in plasma cell bone marrow niches is well-established [9, 27]. However, their role on human B-cell maturation is still controversial [39]. MSC from human bone marrow were shown to inhibit proliferation [40, 41] and B-lymphocyte Ig secretion following polyclonal activation with CpG [42]. In contrast, MSC have been shown to promote LPS-activated B-lymphocyte IgG secretion [43].

Currently, studies on the generation of differentiated B lymphocytes are often based on in vitro interaction with CD154 [44]. These culture models support the differentiation of naive [45–48] or activated B lymphocytes [49–51] upon CD40 activation in the presence of a cocktail of cytokines such as IL-4, IL-10, and IL-6. Such in vitro generated plasma cells are often close to a short-lived phenotype, showing very high levels of Ig secretion and a limited capacity to proliferate. In fact, the majority of in vitro models generate short-lived plasma cells. Conversely, the study of long-lived plasma cells has been quite challenging. Actually, only a few studies have reported the successful generation of long-lived plasma cells in vitro [52, 53]. The goal of this study was to establish culture conditions enabling the differentiation of switched memory B cells into plasma cells using a bone marrow-like environment. For this purpose we used a defined serum-free medium to avoid undesired interactions with animal proteins and to exert maximum control over the components present in the medium. Here, we describe a three-phase culture system which generates high proportions of CD31^+^CD38^+^CD138^+^ plasma cells, using interactions with human bone marrow mesenchymal stem cells.

## 2. Materials and Methods

### 2.1. Isolation of Switched Memory B Lymphocytes

 This study has been approved by Héma-Québec's Research Ethics Committee. Regular platelet donors who agreed to participate in this study have all signed an informed consent. Peripheral blood mononuclear cells (PBMC) from healthy donors were recovered from leukoreduction chambers of plateletpheresis apparatus (Trima Accel, Terumo BCT, Lakewood, CO) and stored frozen as previously described [54]. Switched memory CD19^+^ B lymphocytes were isolated from PBMC by a two-step negative selection using the EasySep™ B-cell Enrichment Kit and EasySep™ custom cocktail removing all IgD- and IgM-positive cells (STEMCELL Technologies, Vancouver, Canada), as previously described [51]. The purity of CD19^+^IgD^−^IgM^−^ intact switched memory B lymphocytes was higher than 95%, as determined by flow cytometry.

### 2.2. Human Mesenchymal Stem Cells

 Mesenchymal stem cells from human bone marrow were obtained from Lonza (Walkerville, MD, USA). Human bone marrow mesenchymal stem cells (MSC) were cultured in *α*-MEM from Sigma-Aldrich (Oakville, ON, Canada) with 10% fetal bovine serum from Life Technology (Burlington, ON, Canada) and 2 mM glutamax from Thermo Scientific (Rockford, IL, USA). A master bank was prepared by expanding the cells for 7 days using a Quantum Cell Expansion System from Terumo BCT (Lakewood, CO, USA). The MSC population was homogenuous, with >95% of the cells of phenotype CD73^+^CD90^+^CD105^+^, as determined by flow cytometry [55].

### 2.3. CD154^+^ Murine Cell Line and Human Eosinophilic Cell Line

L4.5 cells are modified L929 mock cells expressing CD154 at their surface [56]. These adherent cells were cultured in Iscove's Modified Dulbecco's Medium (IMDM) supplemented with 5% low IgG fetal bovine serum (FBS), both from Life Technologies. L4.5 cells were irradiated with 7500 rad using a Gammacell 1000 Elite ^137^Cs-*γ*-irradiator (Nordion International, Kanata, Canada) to prevent their proliferation [57]. The human eosinophilic cell line EOL-1 was obtained from DSMZ (ACC 386; Braunschweig, Germany). They were cultured in Roswell Park Memorial Institute (RPMI) medium with 10% low IgG FBS, from Life Technologies. After 10 days of culture, EOL-1 cells were activated for 9 days with 0.1 mM dibutyryl cyclic adenosine monophosphate (dbcAMP) from Sigma-Aldrich.

### 2.4. Culture of Human CD40-Activated B Cell

#### 2.4.1. Components of Culture Media

Switched memory B lymphocytes were cultured in two different media. The first one, termed FBS, was made of IMDM, 10% ultra-low IgG fetal bovine serum (FBS), and 1% ITS (10 *μ*g/mL human insulin, 5.5 *μ*g/mL human transferrin, and 6.7 ng/mL sodium selenite), all from Invitrogen (Burlington, ON, Canada). The second medium is a bovine protein-free medium (BPFM) [58]. In addition to the supplements added to IMDM, BPFM was supplemented with 60 ng/mL of long-R3 insulin-like growth factor 1 (IGF-1), 100 *μ*M of Trolox, and 5.5 *μ*g/mL of human holotransferrin from Sigma-Aldrich; Lipid mixture 1 (LP-1), composed of 2 *μ*g/mL arachidonic acid, 10 *μ*g/mL of each of linoleic, linolenic, myristic, palmitic, stearic, and oleic acids, 220 *μ*g/mL cholesterol from New Zealand sheep's wool, 2.2 mg/mL Tween-80, 70 *μ*g/mL tocopherol acetate, and 100 mg/mL Pluronic F-68 from Sigma-Aldrich; 20 *μ*g/mL of low density lipoprotein (LDL) from STEMCELL Technologies; 250 *μ*g/mL of 5% human serum albumin (HSA) from Bayer Inc. (Toronto, ON, Canada); and 10 *μ*g/mL of human recombinant insulin from Life Technologies.

#### 2.4.2. Cytokines and Coculture Conditions

Cytokines were added following a three-step culture protocol. During the expansion phase, 50 U/mL of interleukin 2 (IL-2), 120 U/mL of IL-4, and 20 U/mL of IL-10, all from Peprotech (Rocky Hill, NJ, USA), were added. In these conditions, irradiated L4.5 cells were seeded with switched memory B cells at a ratio of one L4.5 to 5 B lymphocytes (1 : 5) to sustain proliferation for 8 to 12 days [45, 59]. For the 4-day long second phase, called transition phase, 10 U/mL IL-6 (from Peprotech) was added to the medium to induce switched memory B-cell differentiation. In addition, the L4.5 : B-cell ratio was reduced to 1 : 20. The last step, the differentiation phase, was performed in the presence of 10 U/mL IL-6 and 20 U/mL IL-10. Control was performed with L4.5 cells at a constant 1 : 20 ratio. At the end of the culture, irradiated L4.5 cells represent less than 5% of the total number of harvested cells (data not shown).

Assays in the presence of activated EOL-1 cells were achieved at a 1 : 20 ratio with switched memory B lymphocytes in a 6.5 mm polycarbonate transwell with 5 *μ*m pores (Fisher, Pittsburgh, PA, USA). EOL-1 cells were seeded in the top well, while switched memory B lymphocytes were seeded in the bottom well in presence of L4.5 cells at a ratio of 1 : 20. MSC were seeded with B lymphocytes at a 1 : 5 ratio. In transwell conditions, MSC were plated in the top well, with or without activated EOL-1. For direct contact assays, MSC were seeded in Primaria 24 wells (Fisher) 1 hour before the addition of B lymphocytes.

The differentiation phase was performed for 8 days next to the transition phase of cell culture. All conditions tested were run in parallel at 37°C with 5% CO_2_, under atmospheric O_2_ conditions (18%) and normoxic physiologic conditions with 8% O_2_ using a Heracell 150i incubator (Thermo Scientific). For assays at 8% O_2_, media were preincubated for 12 to 24 hours in the absence of cytokines before the start of the culture to reach gas pressure equilibrium. Between each step, switched B lymphocytes were centrifuged at 200 ×g to remove the supernatant containing the previous cytokine cocktail. During the culture process, media were changed every 2 days, and corresponding cell lineages were replaced every 4 days. Cell viability was measured on a NucleoCounter NC-250®, after labeling with AO Solution 18 following the manufacturer's instructions (Chemometec, Allerod, Denmark). IgG and IgA content were measured in the culture supernatant by standard ELISA assay as previously described [51].

### 2.5. Flow Cytometry Analysis

Determination of the phenotype of B lymphocytes was performed every 4 to 8 days by flow cytometry during expansion, transition, and differentiation phases. On day 4, cultured B lymphocytes were analysed to confirm their purity by targeting CD3, CD14, CD19, CD45, CD154, IgA, IgD, IgG, and IgM markers. Viability was assessed following a 30-minute staining protocol using pacific blue succinimidyl ester (PB). Cells were washed with PBS supplemented with 1% FBS and 0.001% NaN_3_ and then stained with the appropriate antibodies listed above. Allophycocyanin- (APC-) conjugated anti-CD3 and anti-IgG and phycoerythrin- (PE-) conjugated anti-IgD and anti-CD154 were obtained from BD Biosciences (San Jose, CA, USA). Fluorescein isothiocyanate- (FITC-) conjugated anti-CD14, anti-IgM, and PE-conjugated anti-CD45 were from eBiosciences (San Diego, CA, USA) and FITC-conjugated anti-IgA was from Bio-Rad (Mississauga, ON, Canada). To assess their differentiation status, cells were stained with antibodies targeting CD19, CD31, CD38, CD39, and CD138 markers. MSC were monitored using CD105. FITC-conjugated anti-CD31 (clone WM59), APC-conjugated anti-CD39, and eFluor450-conjugated anti-CD38 (clone HB7) were all from eBiosciences. PE-conjugated anti-CD138 (clone BA-38) was from Abcam (Toronto, ON, Canada), PE-Cy7-conjugated anti-CD105 (clone 43A3) from BioLegend (San Diego, CA, USA), and PE-Cy7-conjugated CD19 from BD Biosciences. Dead cells were stained with 7-aminoactinomycin D (BD Biosciences). Data analyses were performed directly after labeling with a Partec CyFlow ML flow cytometer (Swedesboro, NJ, USA) and FlowMax 3.0 software (Partec, Münster, Germany). For each experiment, dead cells were excluded from analysis, and at least 10 000 events were recorded. Analyses were performed with FCS Express 4 software (De novo, Los Angeles, CA, USA), and positivity for each marker was determined by fluorescence minus one (FMO). In addition, B-cell proliferation was measured with CellVue Burgundy, following the manufacturer instructions (eBioscences) for a period of 4 days. An initial measure performed on day 0 was used as a reference for the assay.

### 2.6. Redox Potential Assessment

The oxidation-reduction potential in culture medium was measured with a micro-Pt/AgCl combination MI-800/410 cm redox electrode (Microelectrodes, Inc., Bedford, NH, USA) connected to a pHi model 510 pH meter (Beckman Coulter, Mississauga, ON, Canada). Measures were performed directly after medium preparation, and analyses in culture supernatants were done at the end of the cultures.

### 2.7. Quantification of Cytokine Secretion

Cytokines and Immunoglobulins were measured in culture supernatants by a Multi-Analyte Profiling technology using Luminex IS 200 system (Luminex, Austin, TX, USA). Each of the targeted molecules was measured separately on flat-bottom Immulon-II plates using Bio-Plex Pro Human, inflammation Panel 1 APRIL/TNFSF13, SDF1*α* + *β*, IP-10/CXCL10, Th17, IL-21, TGF-*β*1, and TGF-*β*2 (all from Bio-Rad). Bio-Plex Pro Reagent kit III was used for APRIL, CXCL12, and CXCL10, Bio-Plex Pro Reagent kit II was used for IL-21, and Bio-Plex Pro Reagent kit I for TGF-*β*. IgA, IgE, IgM, and IgG subclasses were measured using Bio-Plex Pro Human isotyping kit (Bio-Rad). All analyses were done following the manufacturer's instructions.

### 2.8. Spanning-Tree Progression Analysis of Density-Normalized Events (SPADE) Analysis

In order to visualize cellular heterogeneity from a single cell cytometry assay, data for plasma cells (day 8) were run on the SPADE analysis software (Cytobank, Inc, http://www.cytobank.org/) [60]. Analyses were performed on viable cells, classified in 50 nodes, with each one gathering cells according to the similarities in their phenotype. Baseline was set with L4.5 at 21% O_2_ conditions, for each cell culture. Data provided represent the mean of global medians used for the standardization of results from each experiment, and the color scale was set to asymmetric.

### 2.9. Statistical Analysis

All statistical analyses were done using GraphPad InStat version 3 (GraphPad Software, San Diego, CA, USA). Data are presented as means ± S.E.M. The post hoc tests used are described in the appropriate Section 3.

## 3. Results

### 3.1. Proliferation of Switched Memory B Lymphocytes in FBS or Serum-Free Media

**Figure 1:**
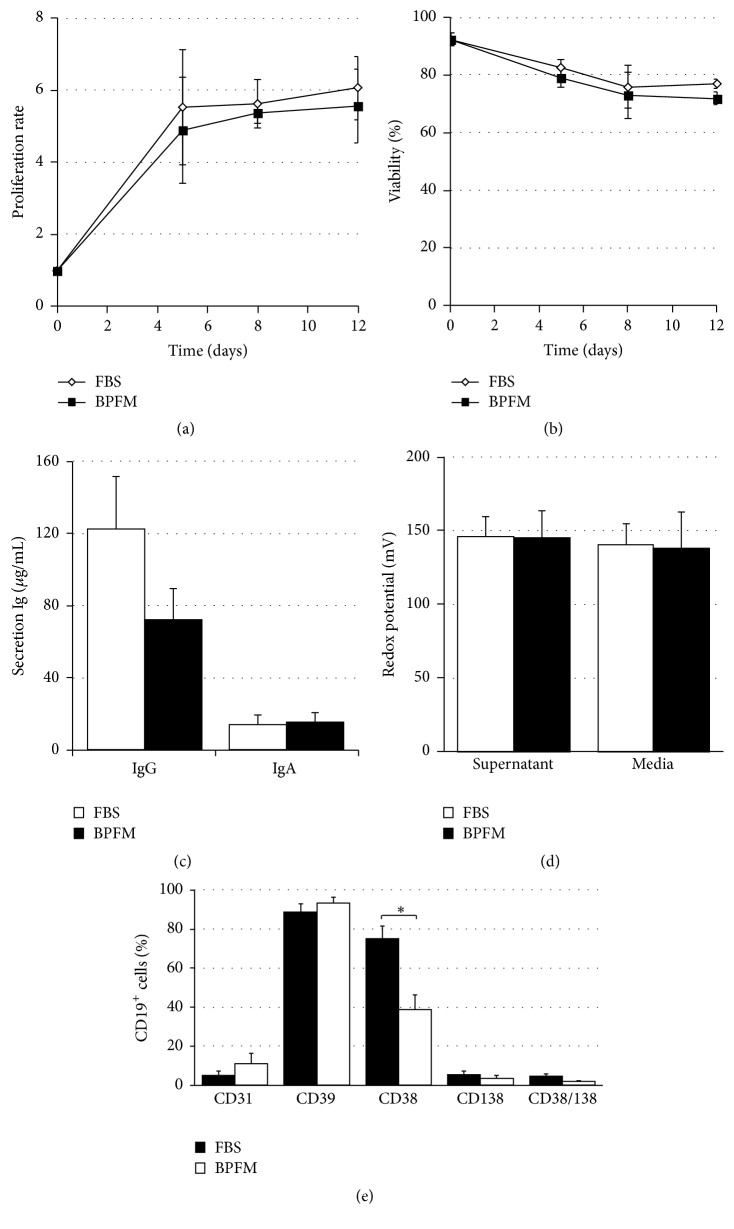
In vitro expansion of switched B lymphocytes in medium containing FBS or in serum-free medium. Five samples of switched memory B lymphocytes were cultured in the presence of IL-2, IL-4, and IL-10 and CD154^+^ cells (L4.5 cells) at a ratio of five B lymphocytes per L4.5. Cells were cultured in parallel in medium containing FBS or in serum-free medium. (a) and (b) show, respectively, the proliferation rate and viability of B lymphocytes after 12 days of culture. (c) The cells were kept in culture for 9 supplemental days to determine the secretion level of IgG and IgA. (d) Redox potential of cell culture supernatants (day 8) and media was measured on 5 independent samples. (e) The proportions of cells expressing CD38 and CD138 markers were determined on gated CD19^+^ B lymphocytes on day 12 (*∗* indicates *p* ≤ 0.05, unpaired Student's *t*-test). All data are from 5 independent experiments, except for the monitoring of CD31 and CD39 in (e), which was done on 4 independent samples. Data are presented as means ± SEM.

We have previously established that the presence of IL-2, IL-4, and IL-10 allows long-term expansion of CD40-activated switched memory B lymphocytes in the presence of bovine serum [51]. Besides, we reported that our in-house serum-free medium, namely, bovine protein-free medium (BPFM), was able to sustain the proliferation of CD40-activated B lymphocytes, including naive and memory cells, using the same cytokine cocktail [58]. The next steps were done to evaluate the outcome of switched memory B lymphocytes when cultured in BPFM. Purified CD19^+^IgD^−^IgM^−^ cells were cultured in parallel for 12 days in BPFM and in a medium containing 10% FBS in the presence of CD154^+^ L4.5 cells and IL-2, IL-4, and IL-10 (Figure 1).

The proliferation rate was calculated for 3- to 4-day periods to monitor the progression of CD19^+^IgD^−^IgM^−^ cells (Figure 1(a)). Overall, during this three-step culture, the cells were expanding similarly in both conditions (Bonferroni multiple comparisons test, *p* > 0.05), giving an average of 5.3 ± 0.2-fold expansion in BPFM and 5.8 ± 0.1-fold expansion in the presence of FBS. Total expansion, starting from 1 × 10^6^ seeded cells, had reached 82- to 429-fold for cells cultured in FBS and 71-to 328-fold in BPFM (data not shown). The presence of FBS was slightly advantageous for the switched activated B lymphocytes in regards to total expansion (paired *t*-test, *p* = 0.0118). Viability assessment did not show any significant differences when comparing both conditions (Figure 1(b)) (Dunn's multiple comparison test; *p* > 0.05), decreasing on day 12 to 77 ± 2% and 72 ± 2% in FBS and BPFM, respectively. The cells were maintained in culture for an additional 9 days to measure their commitment towards differentiation by measuring the secretion of IgG and IgA (Figure 1(c)). IgA secretion was similar in both conditions, reaching 14.4 ± 4.9 *μ*g/mL and 15.2 ± 5.6 *μ*g/mL, in FBS and BPFM, respectively. IgG secretion appeared higher when cells were cultured in the presence of FBS (122.5 ± 28.8 *μ*g/mL) than in BPFM (72.3 ± 17.2 *μ*g/mL); however this difference was not statistically significant (Dunn's multiple comparisons test, *p* > 0.05). The progression towards differentiation was also monitored on day 12 according to CD31, CD38, CD39, and CD138 expression (Figure 1(e)). Overall, the cellular phenotype was similar in both conditions, except for the proportion of CD38^+^ cells, which was lower in cells cultured in BPFM (39% ± 8%, compared to 75% ± 8% in FBS) (unpaired Student's *t*-test, *p* ≤ 0.05). The proportion of CD38^+^CD138^+^ cells was lower than 5% in both conditions. Finally, the measure of redox potential in both media and in cell culture supernatants showed no significant differences (Figure 1(d)).

Overall, we showed that BPFM allows switched memory B lymphocytes to proliferate and to initiate differentiation. This medium was thus used to further investigate the in vitro generation of plasma cells. Noticeably, the significant decrease in the proportion of CD38^+^ cells had no impact on the smaller CD38^+^CD138^+^ cell population.

### 3.2. Differentiation of Switched Memory B Lymphocytes in BPFM under Low Oxygen Levels

**Figure 2:**
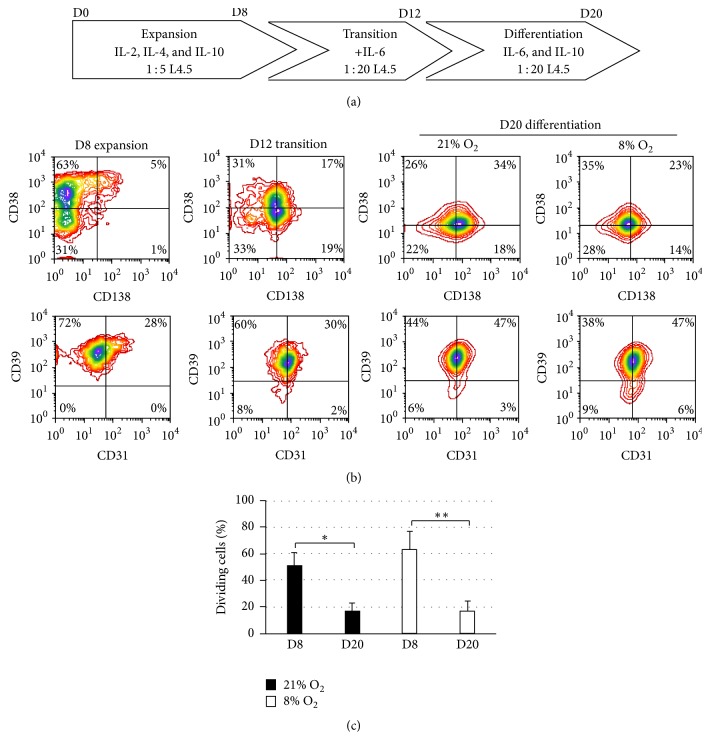
Differentiation profile of switched memory B cells under atmospheric and physiologic O_2_ in BPFM medium. Switched memory B cells were subjected to a 3-step culture process (a). All cultures were done in BPFM. As illustrated in (a), the first 8 days of expansion were done as described in Figure 1. Afterwards, a transition period of four days was applied to decrease the CD154 interaction to a 1 : 20 ratio and to expose B lymphocytes to IL-6. Finally, cells were maintained in the latter condition for an additional 8 days, except that IL-2 and IL-4 were removed from culture medium. During the differentiation phase, cells were subjected in parallel to 21% and 8% O_2_. (b) CD31, CD38, CD39, and CD138 expression were monitored at the end of each phase by gating on viable cells as shown in Figure 3(a). These results are representative of 5 independent experiments. (c) The effect of oxygen levels on cell cycle progression was studied in five additional independent samples cultured as previously mentioned, using the CellVue method. During the last four days of expansion and differentiation phases, cells were subjected to 21% or 8% O_2_ in parallel; (c) show the proportion of dividing cells. Statistical analyses were done using the Dunn multiple comparison *t*-test and the *p* values are *∗* ≤ 0.05 and *∗∗* ≤ 0.01, as indicated. Data are presented as means ± SEM.

B lymphocytes were pushed into differentiation in BPFM using a simple three-step model involving a shift in the L4.5 : B-cell ratio and modifications of cytokines, as previously described [47] (Figure 2(a)). As previously observed, CD38 and CD39 expression rapidly increased following B-cell activation (Figure 2(b), D8). However, CD38 expression decreased during the transition and differentiation steps. This decrease was related to the absence of retinoic acid in the BPFM medium (data not shown), as already reported for CD34^+^ cells [61]. Besides, transition towards differentiation resulted in a slight increase in the number of cells expressing the CD31 and CD138 markers (Figure 2(b)). At the end of the differentiation phase, most of the cells were still positive for CD39 (>85%) and about half of them were also positive for CD31, CD38, and CD138. Overall, subjecting cells to an 8% O_2_ level resulted in phenotypes similar to what is obtained with the standard 21% O_2_ condition. The IgG content appeared higher at 21% O_2_ (73 ± 8 *μ*g/mL) than at 8% O_2_ (25 ± 6 *μ*g/mL). IgA content was similar for both conditions, namely, 10 ± 7 *μ*g/mL and 12 ± 7 *μ*g/mL, respectively, for 21% and 8% O_2_ (data not shown).

Finally, monitoring of cell division using CellVue staining showed evidence for a significant decrease, by about 2- to 3-fold, in the proportion of dividing B lymphocytes during differentiation at 21% and 8% O_2_ (Figures 2(c) and S1 in Supplementary Material available online at http://dx.doi.org/10.1155/2016/7801781). In this assay, cell viability varied from 80 ± 1.5% and 75 ± 1.7% for 21% and 8% O_2_, respectively (data not shown).

Overall, the above results confirm that the BPFM medium is allowing efficient differentiation of B lymphocytes when combined with a low CD154 interaction level in the presence of IL-6 and IL-10. However, at this point, this differentiation appeared independent of oxygen concentration.

### 3.3. Optimization of B-Cell Differentiation

**Figure 3:**
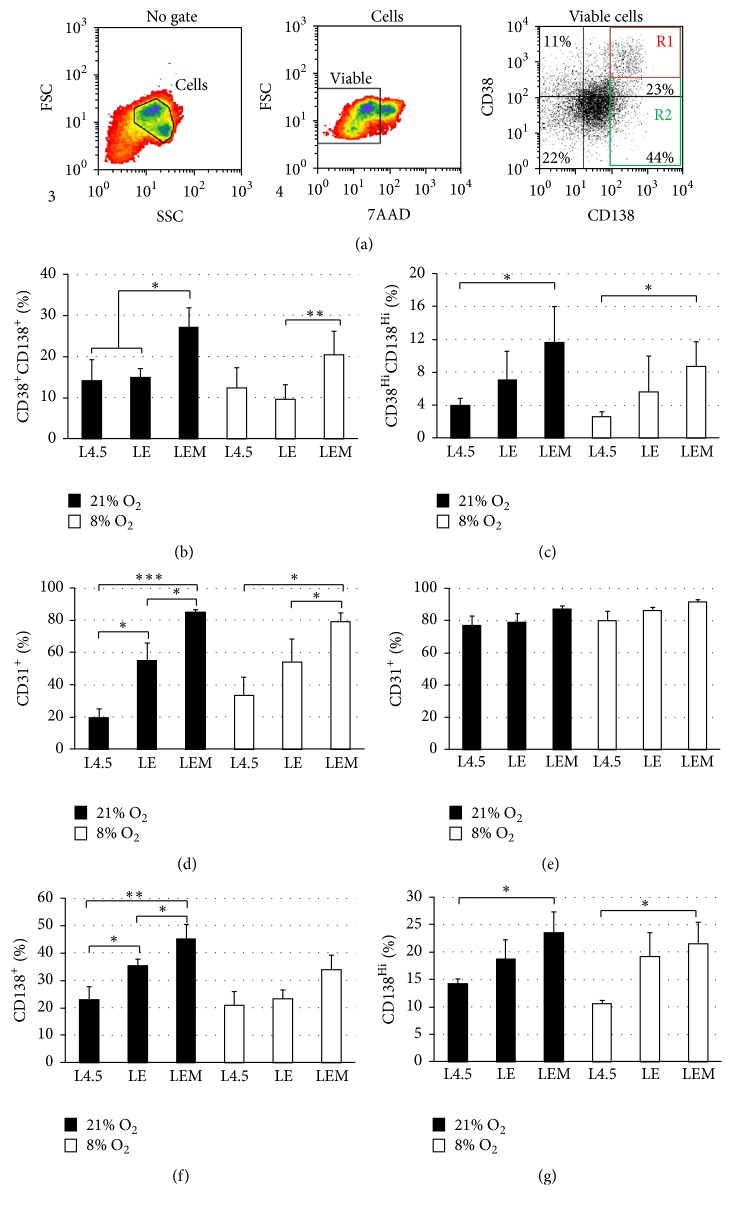
Effects of eosinophil and mesenchymal stem cells on the differentiation of switched memory B lymphocytes. Switched memory B lymphocytes were cultured as described in Figure 2(a) for the expansion and transition phases. Then, cells were exposed to L4.5 cells (L) alone or in combination with EOL-1 cells (E) or EOL-1 cells plus bone marrow MSC (M) during the differentiation phase. During that phase, all cultures were done in 21% and 8% O_2 _condition. (a) Plasma cell content was determined on day 20 by excluding debris and dead cells. Analysis relative to CD38^+^CD138^+^ cells is gated within the upper right (UR) quadrant, and results are shown in (b) and (d). Results for CD38^hi^CD138^hi^ population, gated within R1 (red square), were analysed in (c) and (e). (g) Analysis for CD138^hi^ cells gated within R2 (green square). (b) Frequencies of CD38^+^CD138^+^, (c) CD38^hi^CD138^hi^, (d-e) CD31^+^, (f) CD138^+^, and (g) CD138^hi^ populations are shown for 7 independent experiments for cells cultured in the presence of L4.5 cells alone and for 5 independent experiments for LE and LEM assays. Statistical analyses were done using a Dunn multiple comparison *t*-test with *p* values as indicated: ^*∗*^ *p* ≤ 0.05, ^*∗∗*^ *p* ≤ 0.01, and ^*∗∗∗*^ *p* ≤ 0.005. Data are presented as means ± SEM.

The purpose of the next experiment was to model the environment found in the bone marrow, using an activated eosinophil cell line, namely, EOL-1, and human bone marrow mesenchymal stem cells, during the differentiation phase (Figure 3). For these assays, B lymphocytes were maintained in direct contact with L4.5 cells (L), while EOL-1 (E) and/or MSC (M) were added in the upper chambers of transwell plates.

The proportions of CD38^+^CD138^+^ cells, CD38^hi^CD138^hi^ cells, and CD31^+^ cells within these subpopulations of plasma cells were determined in viable cells at the end of the differentiation phase, on day 20 (Figure 3(a)). No significant differences were observed in total cell numbers within cultures done at 8% O_2_ and 21% O_2_ for all combinations tested. In contrast, the proportions of CD38^+^CD138^+^ cells and CD38^hi^CD138^hi^ cells were greatly influenced by the composition of the coculture partners (Figures 3(b)-3(c)). Differentiating cells subjected to 21% O_2_ showed that CD154 activation alone or in combination with EOL-1 cells allowed the formation of, respectively, 13.9 ± 3.5% and 14.8 ± 2.3% of CD38^+^CD138^+^ cells. In this condition, the addition of MSC to CD154 and EOL-1 cells (LEM) significantly increased the proportion of CD38^+^CD138^+^ to 27.1% ± 4.4% (Dunn comparison *t*-test with *p* ≤ 0.01). When cells were subjected to 8% O_2_, no differences were observed between the addition of EOL-1 to L4.5 cells and L4.5 cells alone (9.6 ± 0.6% and 12.4 ± 2.5%), while a significant increase was observed when MSC were added (Dunn comparison *t*-test with *p* ≤ 0.05). Furthermore, CD154 activation alone allowed the generation of 3,4  ± 0,8% CD38^hi^CD138^hi^ cells, which was significantly less than activation with LEM (11.6 ± 4,3%, *p* ≤ 0.05) (Figure 3(c)). The presence of EOL-1 cells and MSC also resulted in a significant enhancement of differentiation in regards to the proportion of CD31^+^ cells for cultures done at either 21% or 8% O_2_ within the CD38^+^CD138^+^ cell population. Furthermore, the effect was more striking at 21% O_2_, since the proportion of CD31^+^ cells increased to 55.2 ± 10.4% with EOL-1 alone and to 85.1 ± 1.4% when both cells were added to the upper wells (*p* ≤ 0.05) (Figure 3(d)). In contrast, the expression level of CD31 within the CD38^hi^CD138^hi^ cells was systematically higher than 75% in all conditions tested (Figure 3(e)). Finally, the total populations of CD138^+^ and CD138^hi^ cells were favored when MSC were added to EOL-1 and L4.5 cells, increasing by almost 2-fold at 49.4 ± 4.7% (21% O_2_) and 23.7 ± 2.6% (8% O_2_), respectively, when compared to L4.5 cells alone (23.2 ± 5.2% (21% O_2_) and 13.1 ± 2.9% (8% O_2_), Figures 3(f) and 3(g)).

Overall, these results suggest that MSC appear to enhance differentiation of switched memory B lymphocytes. MSC appear to be able to secrete factors which synergize with CD154 stimulation to increase the proportion of plasma cells.

### 3.4. Effect of Mesenchymal Stem Cells on Plasma Cell Differentiation

**Figure 4:**
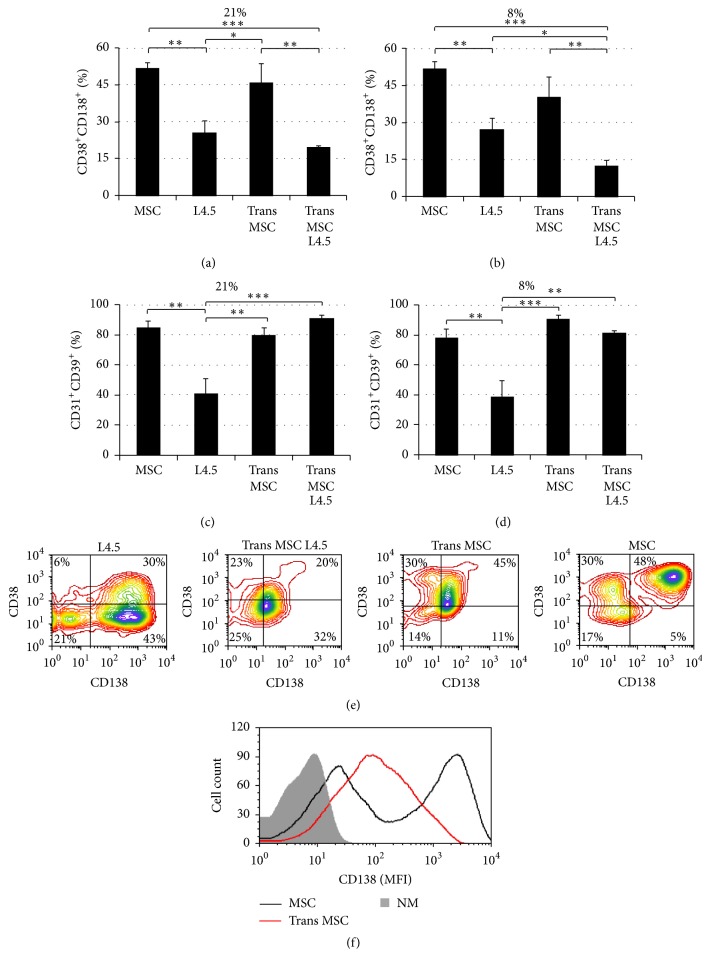
Mesenchymal stem cells influence plasma cell formation with or without cellular contact. Five independent samples of switched memory B lymphocytes were cultured as described in Figure 2(a) for the expansion and transition phases. For the differentiation phase, cells were grown in the presence of L4.5 cells, with or without MSC, which were added either in direct contact or in the upper chambers of a transwell. All assays were done in parallel for all conditions and at 21% and 8% O_2_. The same gating strategy was used as described in Figure 3. (a) and (b) Frequencies of CD38^+^CD138^+^ populations as observed following culture at 21% and 8% O_2_, respectively. (c) and (d) Frequencies of CD31^+^CD39^+^ populations as observed following culture at 21% and 8% O_2_, respectively. (e) A representative profile of CD38 and CD138 expression at the end of the differentiation step for each condition tested at 21% O_2 _is shown. (f) CD138 expression on cells following cultures with MSC in transwell (red line) and in direct contact (gray line), compared to unlabeled cells (shaded). Statistical analyses were done using a Dunn multiple comparison *t*-test, and *p* values are shown as indicated; ^*∗*^ *p* ≤ 0.05, ^*∗∗*^ *p* ≤ 0.01, and ^*∗∗∗*^ *p* ≤ 0.001. Data are presented as means ± SEM.

Since the addition of MSC to switched memory B-cell cultures seems to promote the emergence of plasma cells, we have measured the influence of MSC using direct contact and transwells in the presence or absence of L4.5 cells (Figure 4). The outcome of the cells was monitored as above, using 8% and 21% O_2_ levels. Analyses excluding residual MSC were done on viable cells. The proportions of CD38^+^CD138^+^ cells (Figures 4(a) and 4(b)) and CD31^+^CD39^+^ cells (Figures 4(c) and 4(d)) were evaluated for all conditions. For the four coculture conditions tested, no differences were observed between the two oxygen levels. The addition of MSC in direct contact or in indirect contact using a transwell plate was the most potent stimulus for the emergence of CD38^+^CD138^+^ cells. A direct interaction of switched memory B lymphocytes with MSC allowed the differentiating cells to reach about 50% of the cultured cells, which was significantly higher than with L4.5 cells alone (Figure 4(a)). Furthermore, when MSC were added in the upper chambers of transwells, the proportions of CD38^+^CD138^+^ were comparable to those obtained when in direct contact, reaching 40.2% ± 8.2% and 45.7% ± 7.9% in 8% and 21% O_2_, respectively. However, the expression of CD138 was much higher, according to MFI, when MSC were in direct contact when compared to the transwell setting (Figure 4(f)). Overall, an increase of at least 20-fold of CD138 MFI was observed in 5 independent experiments. Besides, when L4.5 cells were present in the lower chambers, we noticed a significant decrease, by about 2-fold, in the proportions of CD38^+^CD138^+^ cells (Figures 4(a), 4(b), and 4(e)), suggesting an inhibitory effect of L4.5 cells.

On the other hand, the proportion of CD31^+^CD39^+^ cells reached at least 80% and significantly increased in all conditions whenever MSC were present, with or without direct contact with B lymphocytes (Figures 4(c)-4(d)). Overall, a 2-fold increase was noticed compared to cocultures done with L4.5 cells alone, which yielded about 40% CD31^+^CD39^+^ cells at either 8% or 21% oxygen. For this cellular phenotype, no negative effect of L4.5 cells, present in the lower chamber, was observed when MSC were added to the upper compartment of the transwell.

Cocultures of switched memory B lymphocytes were also done with activated EOL-1 cells alone and compared to L4.5 cells alone, as well as the above conditions with MSC. Our results from two independent samples showed that EOL-1 cells were unable to maintain the viability of B lymphocytes. Viability was lower than 40% ± 1.2% and decreased rapidly from day 12 to day 20. Nevertheless, we also noticed that the proportion of residual cells being able to differentiate into CD38^+^CD138^+^ was much lower than that observed with L4.5 and MSC (data not shown).

These observations indicate that MSC alone efficiently promotes the differentiation of switched memory B cells into plasma cell, independently of oxygen concentration. Our results also indicate that MSC could secrete soluble factors contributing to this differentiation-promoting effect. Finally, direct contact with MSC generated plasma cells showing a higher level of CD138 expression.

### 3.5. Increase in FSC^hi^SSC^lo^ Cell Subset following Differentiation in the Presence of MSC

**Figure 5:**
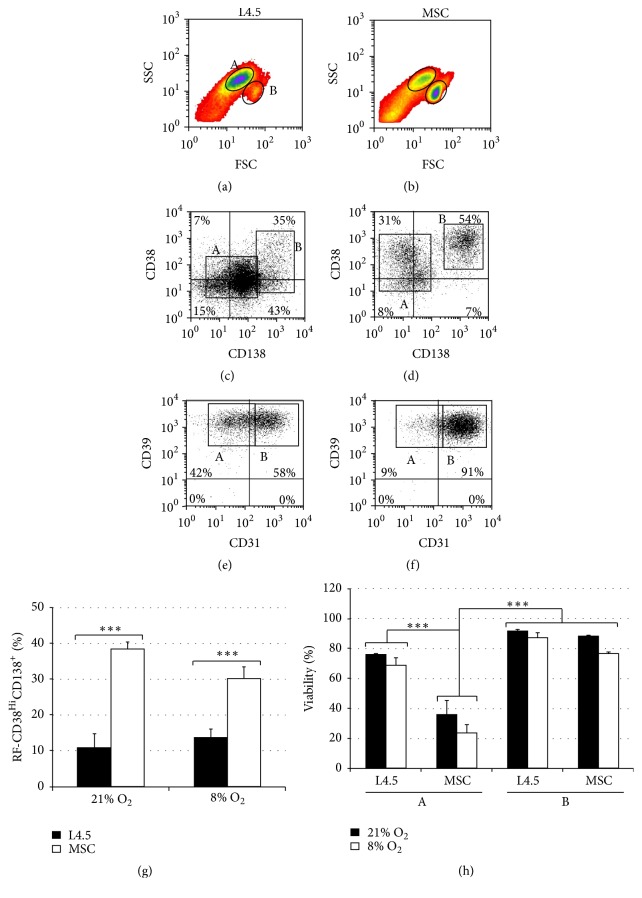
Two subsets of differentiated B lymphocytes can be distinguished according to their size and granularity. Switched memory B lymphocytes were cultured as described in Figure 2(a) during the expansion and transition phases. For the differentiation phase, cells were grown in the presence of L4.5 cells or MSC. All assays were done in parallel at 21% and 8% O_2 _and analyses were done on day 20. (a) and (b) Profiles of populations A and B as revealed by size (FSC) and granularity (SSC) parameters. (c) and (d) Expression of CD38 and CD138 and (e) and (f) expression of CD31 and CD39 were determined and corroborated with populations A and B. Profiles shown in (c)–(f) are representative of five independent samples. (g) Relative frequencies (RF) of CD38^high^CD138^+^ were determined for B populations using this formula: RF = B ÷ (A + B). (h) Viability in A and B populations is shown for all conditions tested. Results in (g) and (h) are presented as the mean ± SEM of five independents experiments. Statistical analyses were done using Dunn multiple comparison *t*-test with ^*∗∗∗*^ *p* ≤ 0.001.

The above results established that MSC stimulation enhances plasma cell generation under either 8% or 21% O_2_ levels. Furthermore, analyses of the cell profiles according to the FSC and SSC parameters also revealed two distinct populations (Figure 3(a)). We thus further characterized these subsets by delineating the FSC^lo^ SSC^hi^ as population A and FCS^hi^SSC^lo^ as population B, as indicated by the respective gates shown in Figures 5(a) and 5(b). All analyses were done at the end of the differentiation phase, and cells were cultured in direct contact with MSC (Figures 5(b), 5(d), and 5(f)) or L4.5 cells (Figures 5(a), 5(c), and 5(e)) and subjected to both oxygen conditions. Overall, about 50% of all cells analysed were present in population B (FCS^hi^SSC^lo^) when the cells were cultured with MSC. However, the frequency of FCS^hi^SSC^lo^ was significantly lower in the presence of L4.5 cells, reaching about 10% and 15% in 8% and 21% O_2_, respectively (Dunn's multiple comparisons test; *p* < 0.05; data not shown).

Additionally, we have observed important variations in regards to the expression of CD31, CD38, CD39, and CD138 within the A and B populations. These variations were independent of the coculture conditions, namely, L4.5 cells or MSC, and oxygen levels (Figure 5 and Table 1). The cells present in populations A and B were all positive for CD39. Overall, population A cells showed low expression of CD31, CD38, and CD138 (Figures 5(c)–5(f)) and low frequencies of cells positive for these markers (Table 1). In contrast, cells forming population B were characterized by a high expression of CD31, CD38, and CD138, which are hallmarks of plasma cells. Furthermore, cells found in gate B included the highest proportion of CD138^+^ cells (≥75%) and CD38^hi^CD138^hi^ cells (85 ± 1.2%, data not shown) when cultured in the presence of MSC (Table 1). To highlight this shift from A to B, we then compared the frequency of CD38^hi^CD138^+^ populations inside each gated subset compared to their content in the total (A + B) population (Figure 5(g)). Again, no significant differences were observed between 8% and 21% O_2_ culture conditions. The proportions of FSC^hi^SSC^lo^CD38^hi^CD138^+^ cells (B population) reached 38.4% ± 1.9% and 30.1% ± 3.2% of total CD38^hi^CD138^+^ cells, following culture in the presence of MSC at 21% and 8% O_2_, respectively. Culturing cells in the presence of L4.5 cells led to lower proportions of plasma cells, as only 10.9% ± 3.9% and 13.7% ± 2.4% of CD38^hi^CD138^+^ cells were observed at 21% and 8% O_2_, respectively (*p* ≤ 0.001 in both conditions). Moreover, a comparison of viabilities in regards to A and B populations (Figure 5(h)) showed that the FSC^hi^SSC^lo^ cells were characterized by the highest viability when cultured with MSC or L4.5 cells. However, low viability was observed for cells in population A when cultured with MSC, with 35.6% ± 9.6% and 23.7% ± 53% viable cells for 21% and 8% O_2_, respectively. In contrast, cells in populations A were still viable at 70% when cultured with L4.5 cells.

We also observed that the CD138^+^ cells generated at the end of the differentiation phase were characterized by low expression of CD19 and were also predominantly positive for CD49d (VLA-4), which is very important for interactions with the bone marrow cellular matrix (data not shown) [62]. Finally, intracellular levels of expression of *κ*/*λ* light chains were verified in three independent experiments for CD138^−^ and CD138^+^ cells corresponding to A and B populations (data not shown). We observed that all CD138^+^ cells express intracellular *κ* and *λ* light chains, in proportions of 40% and 60%, respectively. However, only 70% of CD138^−^ cells were positive for either *κ* or *λ* light chain expression, suggesting heterogeneity in the A but not in the B population.

Once again, these results emphasize the ability of bone marrow MSC to favor plasma cell generation in BPFM medium, but they do not show any impact related to O_2_ levels. Noticeably, the distinct population characterized by FSC^hi^SSC^lo^ includes a majority of plasma cells with a high viability which are better supported by the presence of MSC.

### 3.6. Heterogeneity among Plasma Cells Generated In Vitro

**Figure 6:**
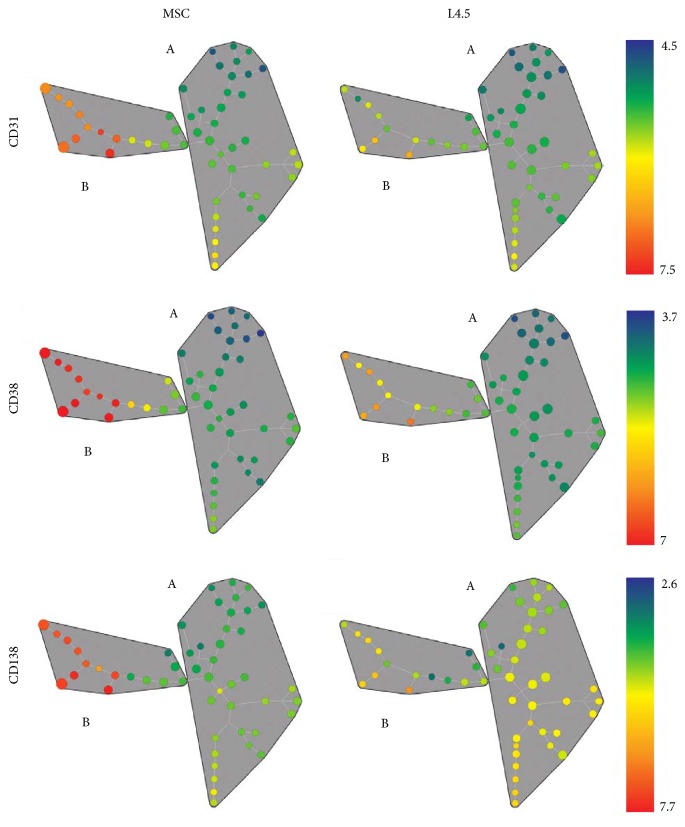
Two-dimensional analysis of differentiated A and B populations. CD31, CD38, and CD138 distributions in cells cultured with MSC and L4.5 cells were analysed on day 20 at the end of the differentiation phase. The strategy used to create SPADE trees was based on size and granularity as clustering channels with 50 nodes. Baseline was set with L4.5 in 21% O_2_ conditions for each cell culture. Results shown are representative of 5 independent experiments. Mean fluorescence intensity ranging from low (green) to high (red) was used to localize CD31, CD38, and CD138 expression as indicated. Color scale was set to asymmetric to highlight differences between the dots.

The above results highlight that the differentiation phase with MSC and L4.5 cells generates two distinct populations of plasma cells. Thus, the heterogeneity of the A and B populations was further investigated using SPADE analysis. This tool enables us to directly visualize the relative expression of CD31, CD38, CD39, and CD138 (Figures 6 and S2). SPADE 2-dimensional representation underlines the distinction of the A and B populations inside gated viable cells. No variations were observed for CD39 expression. The nodes representing population B, namely, FCS^hi^SSC^lo^, are showing higher expression of CD31, CD38, and CD138 when compared to the nodes of population A (Figure 6). We observed, similarly to what was shown above, that MSC were more efficient in maintaining this plasma cell population than L4.5 cells; yet no distinctions were seen according to oxygen levels.

### 3.7. Profiling of Cytokine Secretion by MSC

**Figure 7:**
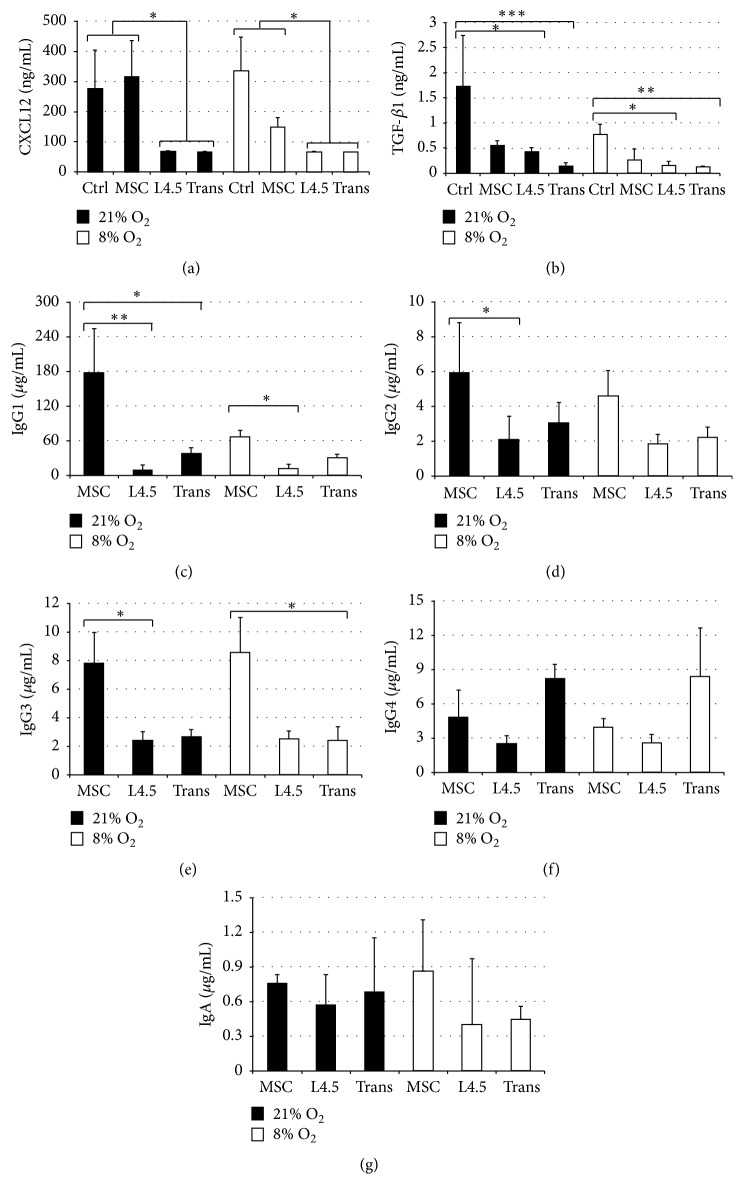
Cytokines and immunoglobulins secretions. All analyses were done at the end of the differentiation phase (day 20). (a and b) CXCL12 and TGF-*β*1 contents were determined in cell culture supernatants of differentiated cells. Control (Ctrl) corresponds to the supernatant of MSC cultured in *α*-MEM for 4 days in the absence of B lymphocytes. All other conditions correspond to B lymphocytes in coculture with MSC or L4.5 cells or B lymphocytes cultured in the presence of MSC which were seeded in the upper chamber of transwell (Trans). Cultures were done in parallel and at 21% or 8% O_2_. (c–g) IgG1, IgG2, IgG3, and IgG4 secretion were determined in cell culture supernatants on day 20. Statistical analyses were performed using a Dunn multiple comparison *t*-test (^*∗*^ *p* ≤ 0.05, ^*∗∗*^ *p* ≤ 0.01, and ^*∗∗∗*^ *p* ≤ 0.001). No significant differences were observed between MSC in direct contact and transwell assays at 8% O_2_ (*p* = 0.0556; Kruskal-Wallis *U*-test). Except for the results of MSC in direct contact, which were obtained from four independent experiments, all data are from five independent experiments and all are presented as means ± SEM.

The observations (Figures 3 and 4) that switched B lymphocytes were directed to differentiate towards plasma cells when cultured in the presence of MSC, even without direct contact, suggested the involvement of soluble factors in the differentiation-promoting effect. Thus, further evaluations of culture supernatants using multiplex Luminex assays were performed to measure their contents in IL-21, CXCL10, CXCL12, TGF-*β*1, TGF-*β*2, and APRIL at the end of the differentiation phase after coculture with L4.5 cells or with MSC, in either direct contact or transwell (Figures 7(a) and 7(b)). Our results revealed that IL-21, CXCL10, TGF-*β*2, and APRIL were in negligible amounts for all tested conditions (data not shown). In fact, we were able to detect only CXCL12 and TGF-*β*1 in culture supernatants, and the same secretion patterns were observed for both O_2_ levels. The quantities of CXCL12 were significantly higher when MSC were in direct contact with the differentiating cells. Curiously, supernatants of MSC added in transwell without contact did not contain comparable amounts of CXCL12 than direct cocultures. We determined that MSC, when cultured alone (Ctrl), can secrete 276 ± 129 ng/mL and 335  ±  112 ng/mL of CXCL12 at 21% and 8% O_2_, respectively. Noticeably, when B lymphocytes were cultured in 21% O_2_ in contact with MSC, the secretion of CXCL12 reached 315 ± 119 ng/mL; this concentration that was reduced by about 50% (148  ±  32 ng/mL) was detected when the culture was done in 8% O_2_.

We observed very high variations among samples for the amount of TGF-*β*1 (Figure 7(b)). However, TGF-*β*1 secretion appeared significantly different only when secretion of MSC alone was compared to that of B lymphocytes cultured in the presence of either L4.5 cells or MSC in transwell assays done at 8% or 21% O_2_. All coculture conditions showed similar quantities of TGF-*β*1 secretion. These results indicate that MSC can efficiently secrete CXCL12 and TGF-*β*1. Furthermore, conditions leading to reduced levels of CXCL12 and TGF-*β*1, for example, in transwell assays or in 8% O_2_, may suggest a variation in the B lymphocytes' capacity to use soluble factors.

### 3.8. Functional Differentiation of Switched Memory B Lymphocytes

The functional characteristics of the differentiated cells were further characterized by evaluating their secretion of immunoglobulins (Figures 7(c)–7(g)). As expected, IgG_1_ was the highest and the main isotype detected in our supernatants for all conditions tested. IgG4 and IgA were detected in all conditions, with no differences between coculture conditions or oxygen levels. No IgM was detected in these supernatants (data not shown). The most significant difference, an almost 10-fold increase, was observed for IgG_1_ secretion by cells cultured in direct contact with MSC compared to those cultured with L4.5 cells, regardless of the oxygen level (Figure 7(c)). However, at 21% oxygen, IgG_1_ secretion was significantly higher when cells were in contact with MSC (66.5 ± 12.9 *μ*g/mL) than in transwells (31.1 ± 7.2 *μ*g/mL). IgG_2_ and IgG_3_ secretion levels were higher when cells were in contact with MSC compared to L4.5 cells in 21% O_2_ (Figures 7(d) and 7(e)).

Furthermore, we have estimated the secretion rate according to total IgG content using the seeding density on day 16. At that point, cells were washed and put back in fresh culture medium for four supplemental days. The estimated secretion rate is 18 pg of IgG per cell per day (data not shown).

Collectively, these data confirm that MSC are very efficient at supporting the generation of functional Ig-secreting cells displaying several characteristics of fully differentiated plasma cells.

## 4. Discussion

In this study, we show that MSC allow the differentiation of human switched memory B cells into CD38^hi^CD138^+^ plasma cells in a serum-free medium. To our knowledge, this is the first model allowing the generation of CD31^+^CD38^hi^CD138^+^ cells in the presence of MSC from human bone marrow and the first report of such differentiation in the absence of bovine serum. In addition, the newly generated plasma cells are mainly nondividing cells and gain Ig-secreting function. The detection of CXCL12 and TGF-*β*1 secreted by MSC validates the usefulness of the in vitro model aiming to reproduce an environment similar to plasma cell niches found in the bone marrow. Our results also highlight the differential effects of CD40 or MSC stimulation on the differentiation of human switched memory B lymphocytes. While MSC favored the commitment towards CD31^+^CD38^hi^CD138^+^ plasma cells, L4.5 cells seem to be slowing down this process and to preferentially activate cells with an intermediate CD31^±^CD38^lo^CD138^lo^ phenotype. Finally, we observed that the generation of newly formed plasma cells appears independent of oxygen levels in our model.

The in vitro generation of human long-lived plasma cells has been reported in two independent studies [52, 53]. Cocco and collaborators were the first to report the long-term maintenance of plasma cells using a three-step culture allowing maintaining cells for up to 62 days [52, 53]. As a first step, they used polyclonal activation of peripheral blood CD27^+^ B cells to generate plasmablasts. The last step was designed to reproduce stromal environment using the gamma-irradiated fibroblastic mouse cell line M2-10B4 supplemented with IL-6 and IL-2. Their model generated CD38^hi^CD138^hi^ cells which were characterized by expression of genes associated with long-lived plasma cells and were secreting IgG at about 150 pg/cell/day. The authors however reported that a rapid decrease in total viable cell numbers was observed during this last step, suggesting that persistent viable cells were not abundant in their model.

A second group also used purified peripheral blood CD27^+^ cells, but in a four-step culture system [52]. Similar to Cocco et al., CD27^+^ cells were first activated through soluble CD40 interaction in the presence of a polyclonal activator (CpG) and a mixture of IL-2, IL-10, and IL-15. Jourdan's group was able to generate long-lived plasma cells using stromal cells purified from human tonsils, in the presence of IL-6 and APRIL. Long-lived plasma cells were generated by purifying CD138^+^ cells obtained at the end of the third step and incubating these cells with stromal cells, with or without contact, for the last culture step. At the end of their 4-step process, Jourdan and collaborators obtained newly formed CD38^+^CD138^+^ cells typical of long-lived plasma cells according to their gene expression pattern. These cells were maintained in culture for months. These authors also observed a rapid decrease in cell numbers during the final step of their culture system.

One unique study reports the differentiation of switched memory B lymphocytes using contact with bone marrow MSC [63]. In their study, Traggiai and collaborators have cultured purified cells with MSC, combined with stimulation by soluble CD40L and CpG. Interestingly, they found that MSC favored IgG secretion when B lymphocytes were in direct contact compared to a barrier system modeled by transwell assays.

Noticeably, all of the above studies were performed with media containing bovine serum and used soluble CD40 and polyclonal activation in a separate first step or simultaneously with other cellular effectors [52, 53, 63]. In our model, the source of B lymphocytes is also peripheral blood, but the selection method we use includes CD27^−^ IgG- and IgA-positive cells [49] and excludes CD27^+^IgD^+^IgM^+^ cells [64], which are, respectively, absent and present in Jourdan and Cocco culture systems. Our culture model was initiated by an 8-day expansion step generating CD40-activated memory B lymphocytes [47, 65]. The expansion rate we obtained was at least 20-fold; that is, starting with 1 × 10^6^ switched memory B lymphocytes on day 0, we could generate up to 20 × 10^6^ cells. Despite the fact that cells stopped to proliferate and viability decreased during the differentiation phase, we were able to recover at least 5 × 10^6^ CD138^+^ cells on day 20. The last step of our culture system was based upon direct contact with viable human bone marrow MSC in the presence of IL-6 and IL-10. We did not maintain the cells for longer periods, as we deliberately stopped cultures on day 20, but the observed phenotype was similar to that reported by Cocco and Jourdan studies. MSC could favor plasma cell formation and survival by secretion of CXCL12 and TGF-*β*1 [66] and direct contact as reported in studies of the bone marrow microenvironment of plasma cells [27]. MSC secretion was probably supported by the presence of IL-6 in our culture medium [67] and could have been increased under low oxygen conditions [68–70]. TGF-*β*1 can favor the quiescent state of hematopoietic stem cells in the bone marrow [71] and thus might also help to maintain the survival of plasma cells. In addition, the newly generated plasma cells were almost all positive for CD31, an adhesion molecule, supporting their commitment to respond to direct cellular interactions.

APRIL is considered essential for plasma cell generation [13, 52]. However, we did not add APRIL in our culture medium and did not detect APRIL in our coculture supernatants using L4.5 cells, EOL-1 cells, or MSC. Such discrepancy with other studies could be linked to the fine composition of the switched memory B lymphocytes used in our study, which included both CD27^+^ and CD27^−^ cells and excluded IgD^+^ and IgM^+^ cells.

In our model, the newly generated plasma cells were secreting approximately 18 pg of IgG per cell per day, which is quite similar to the rate reported by Jourdan et al. for long-lived plasma cells [52]. From a molecular perspective, we can estimate that one cell is able to release an average of 800 IgG molecules per second. The secretion rate of short-lived plasma cells can reach up to 10 000 IgG/sec/cell [2]. Therefore, our observation of a low secretion rate is consistent with a long-lived character for our in vitro generated plasma cells. Furthermore, the absence of benefit for cultivating in 8% O_2_ could be explained by a very low exposure to oxidative stress, which is usually associated with a very high secretion of Ig [4]. Moreover, the direct contact with MSC favored a low IgG secretion and correlates with previous observations done with MSC from bone marrow and B lymphocytes [63, 72].

Further analyses revealed the emergence of two distinct populations, FCS^lo^SSC^hi^ and FSC^hi^SSC^lo^, from switched memory B lymphocytes. Both populations were present regardless of L4.5 cells or MSC stimulation. These observations could be related to the hypothesis of a predefined fate upon differentiation [73] related to asymmetrical divisions in germinal center (GC) reactions, leading to different fates for daughter cells [74]. Such variability in FSC and SSC patterns has already been reported for plasma cell populations in regards to their cytoplasmic composition [75]. Furthermore, we observed that the FCS^lo^SSC^hi^ cells were mainly CD31^−^CD38^lo^CD138^lo^, a phenotype that resembles early plasma cells [76]. Although we have some indications that their secretion capacity could be different from that of FSC^hi^SSC^lo^ cells, this has to be confirmed with sorted A and B cell populations using ELISPOT or ELISA methods.

In conclusion, our study presents a culture model enabling the generation of plasma cells from switched memory B lymphocytes. This model allows the production of large quantities of plasma cells by combining a short expansion step (<14 days) and a differentiation phase in a bone marrow-like environment. All cultures were done in the absence of bovine serum which, in the perspective of cellular therapy development, is very important for preventing adverse immunogenicity [77–79]. We believe that this model opens new avenues for autologous cell therapy for immunocompromised patients following cancer treatment or stem cell transplantation. Such plasma cells generated in vitro from activated switched memory B lymphocytes could attenuate the risk of infections in patients [80]. Clinical studies have showed that 85% of bone marrow transplant patients will have at least one infection during the year that follows the graft, with a higher risk during the first 100 days [81]. Furthermore, in our model, the newly formed plasma cells are exposed to human physiological oxygen levels [82–84], so they could adapt more rapidly and migrate more easily to bone marrow niches. However, additional investigations are needed to confirm the long-lived character of our in vitro generated plasma cells. First, we plan to produce enough cells to detect Blimp1, IRF4, and XBP-1 proteins, as has been reported elsewhere [52, 53, 76]. We are also currently investigating whether plasma cells generated in this model will be able to migrate to the bone marrow and reconstitute the secretion of human immunoglobulins in a NOD-SCID IL2R*γ* (null) mouse model that is primarily designed for in vitro generated stem cell transplantation [85].

## Supplementary Material

Supplementary Material includes two Supplementary Figures. The Supplementary Figures contain CellVue staining to monitor cell division by flow cytometry during expansion and differentiation phases (Supplementary Figure 1) and SPADE profiles of newly-generated plasma cells to visualize heterogeneity following cultures with L4.5 cells and MSC according to CD31, CD38, CD39 and CD138 markers (Supplemental Figure 2).

## Figures and Tables

**Table 1:** Expression of CD31, CD38, CD39 and CD138 among plasma cells population on day 20.

Condition	Population^1^	Cells^2^ (%)			
		CD31	CD38	CD39	CD138
21% O_2_					
L4.5	A	19,2 ± 4,8	23,0 ± 6,8	93,3 ± 1,0	44,9 ± 4,8
	B	57,8 ± 4,4	49,4 ± 8,9	93,1 ± 4,4	57,3 ± 8,0
MSC	A	26,2 ± 4,4	40,8 ± 5,7	77,9 ± 7,6	11,8 ± 6,2
	B	87,4 ± 2,3	86,3 ± 5,6	97,0 ± 2,4	74,9 ± 6,0
8% O_2_					
L4.5	A	14,5 ± 4,6	15,5 ± 4,8	91,6 ± 1,4	24,0 ± 6,4
	B	62,6 ± 2,3	42,7 ± 6,0	97,6 ± 0,9	52,5 ± 7,6
MSC	A	23,9 ± 3,3	36,7 ± 2,4	76,2 ± 5,1	7,3 ± 1,7
	B	87,1 ± 1,7	89,9 ± 1,2	98,2 ± 1,3	78,3 ± 4,1

^1^Analyses were done according to the populations A and B as describes respectively as FSC^Low^SSC^Hi^ and FSC^Hi^SSC^Low^ as showed in Figures 5 and 6.

^2^The proportion of cells positive for each marker was determined for 5 independent experiments by cytometry analysis as showed in Figure 6. Data are mean ± S.E.M.
