# Supplementary figures and images for: Bone Marrow Mesenchymal Stem Cells Enhance the Differentiation of Human Switched Memory B Lymphocytes into Plasma Cells in Serum-Free Medium
Source: J Immunol Res. 2016 Oct 31;2016:7801781. doi: 10.1155/2016/7801781 (PMC5107863; doi:10.1155/2016/7801781)

Supplemental 1

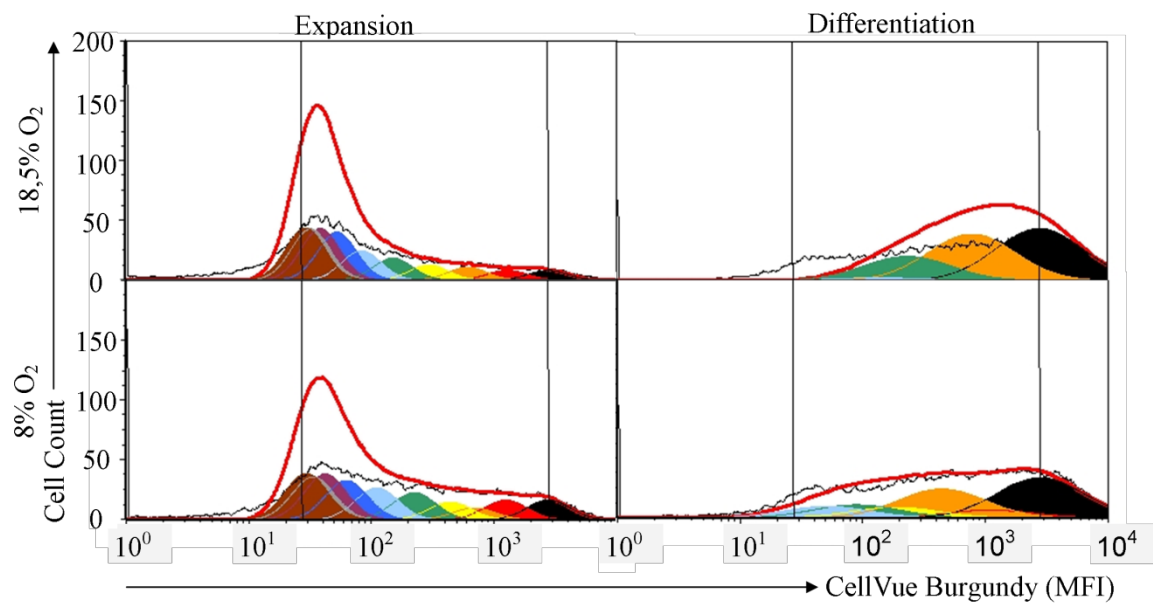

Supplemental 2

A

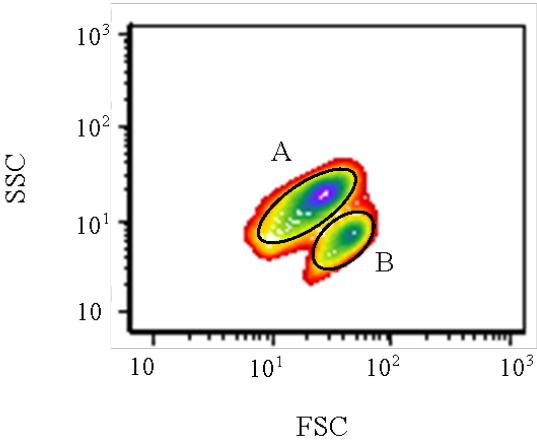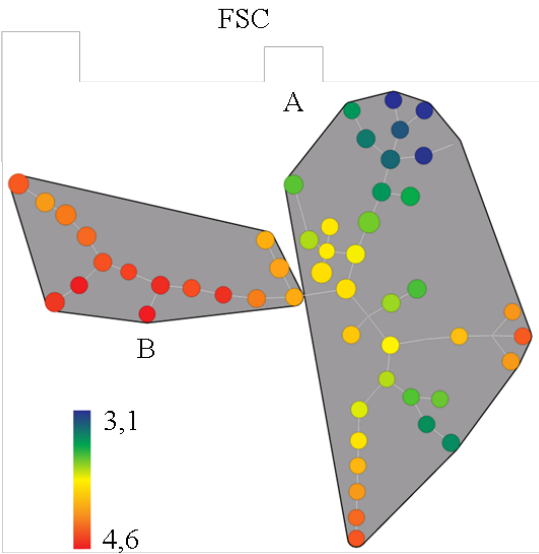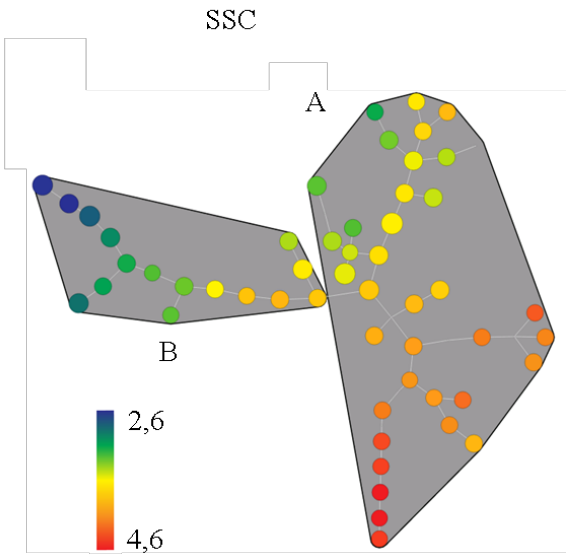

B

CD31

21% MSC

21% L4.5

8% MSC

8% L4.5

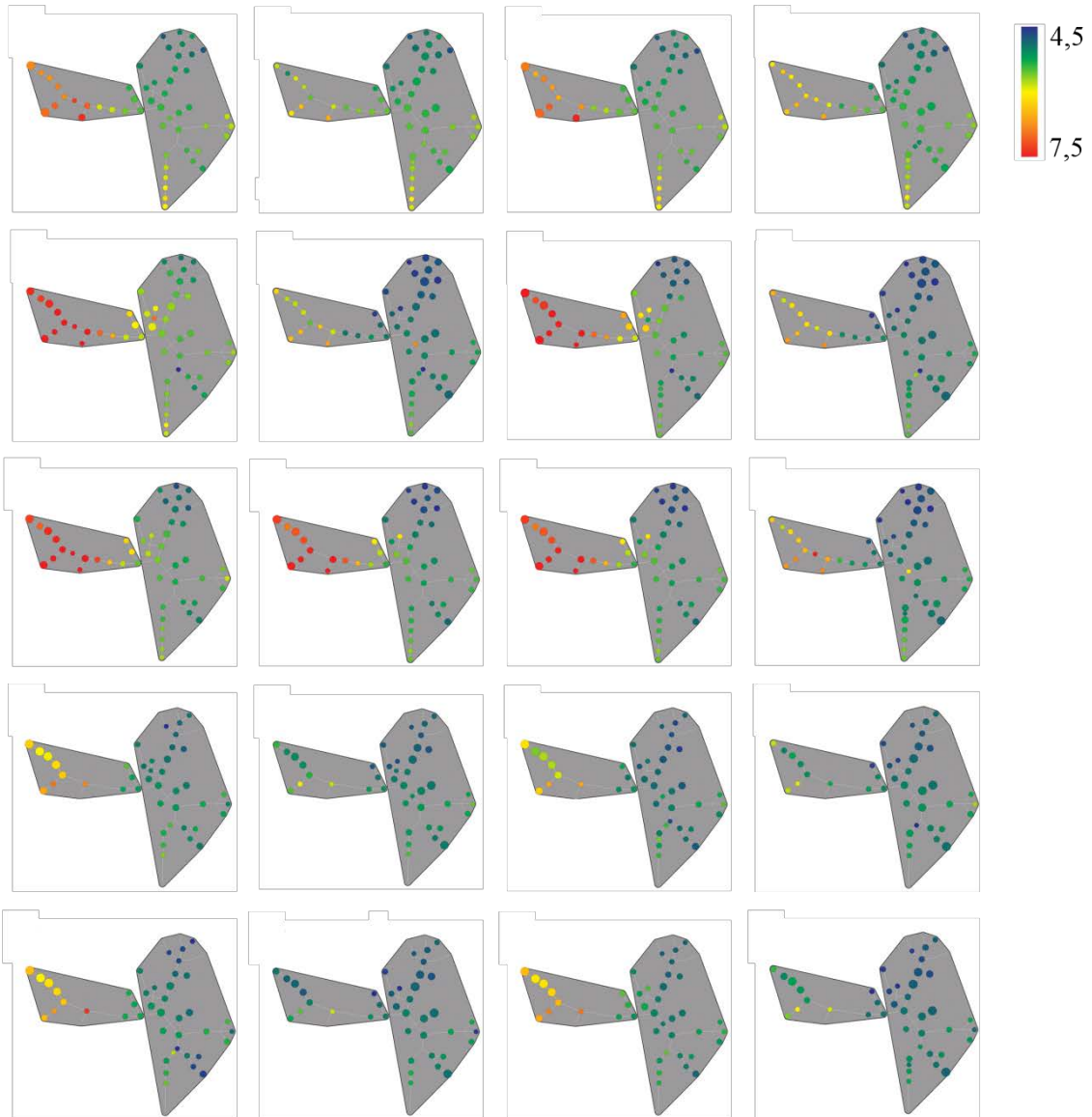

C

CD38

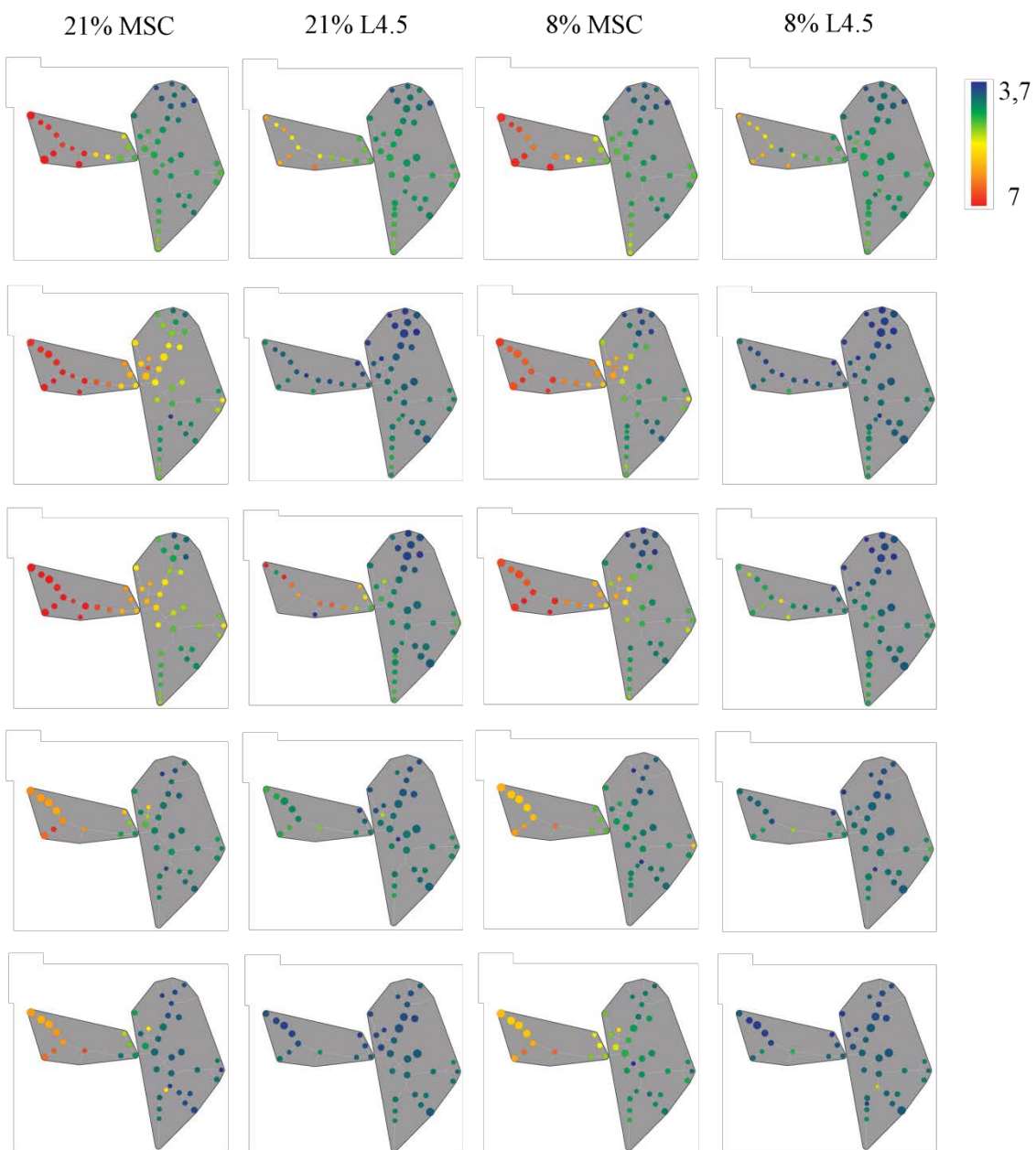

D

CD39

21% MSC

21% L4.5

8% MSC

8% L4.5

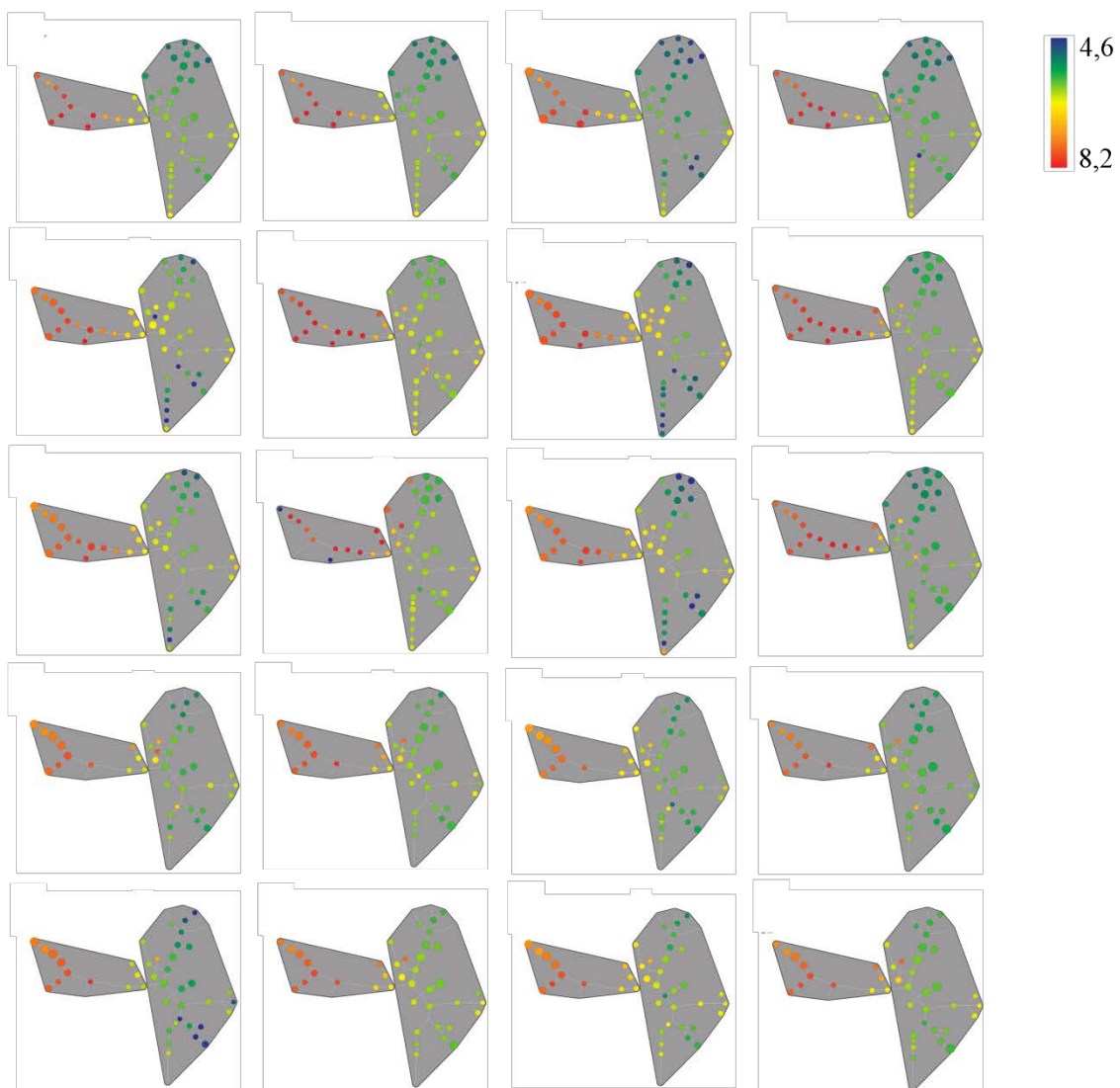

E

CD138

21% MSC

21% L4.5

8% MSC

8% L4.5

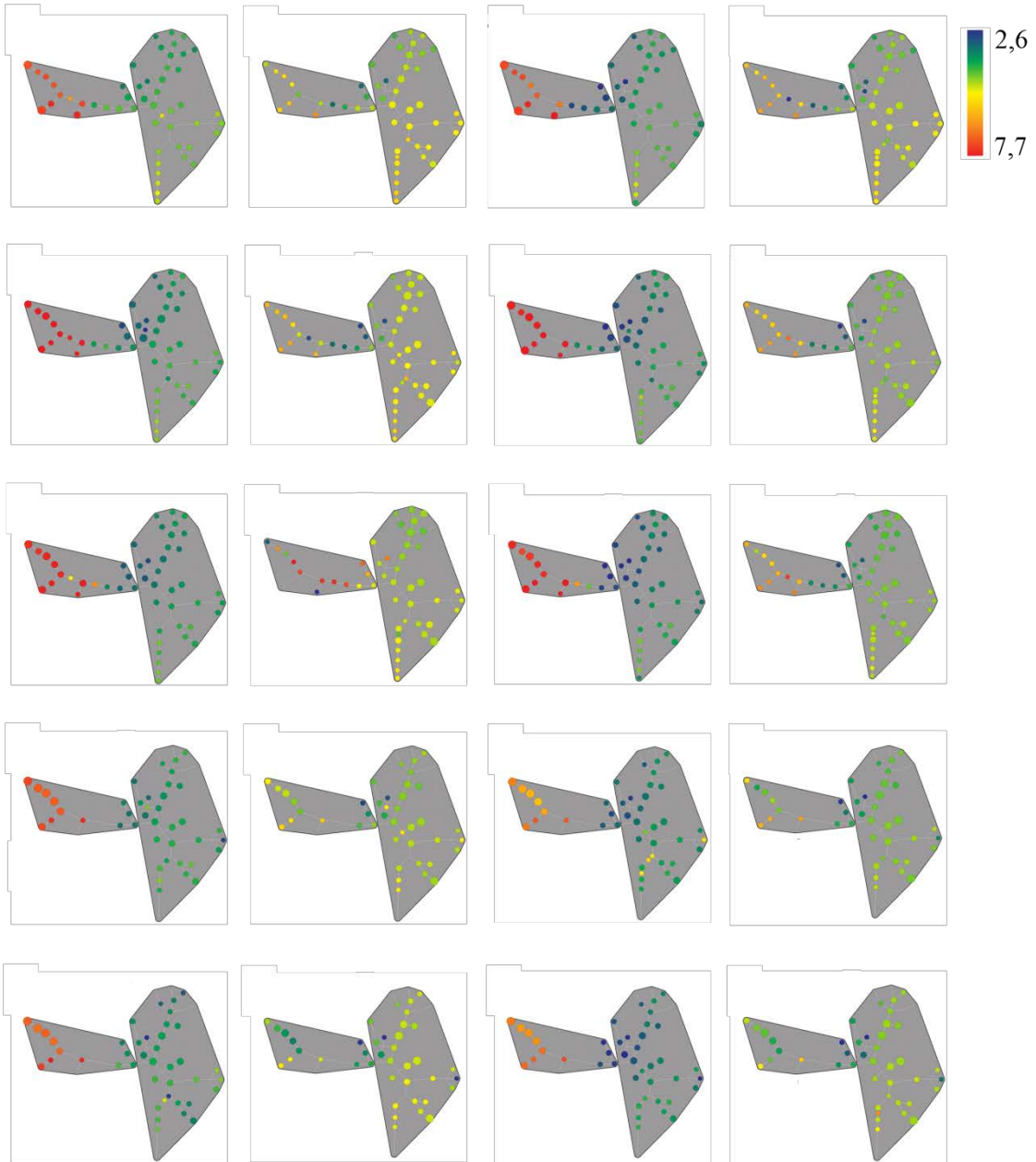

Supplement: Supplementary file 1 — Supplementary Material includes two Supplementary Figures. The Supplementary Figures contain CellVue staining to monitor cell division by flow cytometry during expansion and differentiation phases (Supplementary Figure 1) and SPADE profiles of newly-generated plasma cells to visualize heterogeneity following cultures with L4.5 cells and MSC according to CD31, CD38, CD39 and CD138 markers (Supplemental Figure 2). [file 7801781.f1.pdf]
